# Integrative mapping of preexisting influenza immune landscapes predicts vaccine response

**DOI:** 10.1172/JCI189300

**Published:** 2025-07-15

**Authors:** Stephanie Hao, Ivan Tomic, Benjamin B. Lindsey, Ya Jankey Jagne, Katja Hoschler, Adam Meijer, Juan Manuel Carreño Quiroz, Philip Meade, Kaori Sano, Chikondi Peno, André G. Costa-Martins, Debby Bogaert, Beate Kampmann, Helder Nakaya, Florian Krammer, Thushan I. de Silva, Adriana Tomic

**Affiliations:** 1 Atomic Lab, National Emerging Infectious Diseases Laboratories (NEIDL), Boston University, Boston, Massachusetts, USA.; 2 The Florey Institute of Infection and NIHR Sheffield Biomedical Research Centre, and; 3 Division of Clinical Medicine, School of Medicine and Population Health, University of Sheffield, Beech Hill Road, Sheffield, United Kingdom.; 4 Vaccines and Immunity Theme, Medical Research Council Unit The Gambia at the London School of Hygiene and Tropical Medicine, Fajara, The Gambia.; 5 Respiratory Virus Unit, Public Health Microbiology Reference Services, UK Health Security Agency, London, United Kingdom.; 6 National Institute for Public Health and the Environment, Bilthoven, Netherlands.; 7 Department of Microbiology and; 8 Center for Vaccine Research and Pandemic Preparedness (C-VaRPP), Icahn School of Medicine at Mount Sinai, New York, New York, USA.; 9 Center for Inflammation Research, Institute for Regeneration and Repair, University of Edinburgh, Edinburgh, United Kingdom.; 10 Department of Clinical and Toxicological Analyses, School of Pharmaceutical Sciences, University of São Paulo, São Paulo, Brazil.; 11 Micromanufacturing Laboratory, Institute for Technological Research, São Paulo, Brazil.; 12 Department of Pediatric Infectious Diseases and Immunology, UMC Utrecht-WKZ, Utrecht, Netherlands.; 13 Vaccines and Immunity Theme, London School of Hygiene and Tropical Medicine, London, United Kingdom.; 14 Charité Centre for Global Health, Berlin, Germany.; 15 Hospital Israelita Albert Einstein, São Paulo, Brazil.; 16 Ignaz Semmelweis Institute, Interuniversity Institute for Infection Research, Medical University of Vienna, Vienna, Austria.; 17 Department of Pathology, Molecular and Cell-Based Medicine, Icahn School of Medicine at Mount Sinai, New York, New York, USA.; 18 Department of Virology, Immunology & Microbiology, Boston University Chobanian and Avedisian School of Medicine, Boston, Massachusetts, USA.; 19 Biomedical Engineering, Boston University, College of Engineering, Boston, Massachusetts, USA.

**Keywords:** Clinical Research, Immunology, Virology, Adaptive immunity, Influenza, Vaccines

## Abstract

**BACKGROUND:**

Predicting individual vaccine responses is a substantial public health challenge. We developed Immunaut, an open-source, data-driven framework for systems vaccinologists to analyze and predict immunological outcomes across diverse vaccination settings, beyond traditional assessments.

**METHODS:**

Using a comprehensive live attenuated influenza vaccine (LAIV) dataset from 244 Gambian children, Immunaut integrated prevaccination and postvaccination humoral, mucosal, cellular, and transcriptomic data. Through advanced modeling, our framework provided a holistic, systems-level view of LAIV-induced immunity.

**RESULTS:**

The analysis identified 3 distinct immunophenotypic profiles driven by baseline immunity: (a) CD8^+^ T cell responders with strong preexisting immunity boosting memory T cell responses; (b) mucosal responders with prior influenza A virus immunity developing robust mucosal IgA and subsequent influenza B virus seroconversion; and (c) systemic, broad influenza A virus responders starting from immune naivety who mounted broad systemic antibody responses. Pathway analysis revealed how preexisting immune landscapes and baseline features, such as mucosal preparedness and cellular support, quantitatively dictate vaccine outcomes.

**CONCLUSION:**

Our findings emphasize the power of integrative, predictive frameworks for advancing precision vaccinology. The Immunaut framework is a valuable resource for deciphering vaccine response heterogeneity and can be applied to optimize immunization strategies across diverse populations and vaccine platforms.

**FUNDING:**

Wellcome Trust (110058/Z/15/Z); Bill & Melinda Gates Foundation (INV-004222); HIC-Vac Consortium; NIAID (R21 AI151917); NIAID CEIRR Network (75N93021C00045).

## Abstract 2

<span>Using one of the most comprehensive LAIV immune datasets compiled to date, *immunaut*, an integrative machine learning framework, identifies distinct immunophenotypic responder groups shaped by baseline immune landscapes, advancing precision vaccinology and guiding more effective, personalized immunization strategies.</span>

## Graphical abstract

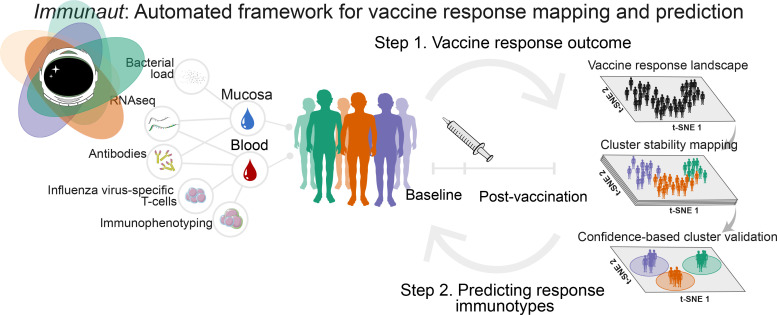

## Introduction

Understanding the ability of a vaccine to elicit an effective immune response (i.e., vaccine immunogenicity), is fundamental to guiding vaccination programs (1). Traditional evaluation methods often measure humoral and cellular immunity in isolation, overlooking their intricate interplay (2–4). Although emerging high-dimensional profiling technologies enable more holistic assessments (3), comprehensive evaluations that simultaneously capture systemic, mucosal, and cellular immune responses remain rare. This poses a substantial barrier to predict vaccine-induced immunity, especially for complex vaccines such as the live attenuated influenza vaccine (LAIV), which engages multiple arms of the adaptive immune system (5, 6).

Here, we bridge this gap by leveraging an extensive LAIV immune dataset from a cohort of 244 children aged 24–59 months in The Gambia enrolled in a phase 4 immunogenicity study (7–9). We integrated humoral, cellular, and mucosal responses with detailed baseline clinical and immunological measurements. Our dataset includes multiple serum antibody and T cell responses, mucosal IgA, transcriptomic profiles, and microbiological assessments, providing the statistical power to delineate robust immunogenicity patterns and advance a systems-level understanding of vaccine response.

Translating such multifaceted data into actionable insights is challenging. Predictive methods must account for high interindividual variability and incorporate baseline immune features from limited samples (10–14). Existing analytical tools have begun integrating diverse data types (15–18), but to our knowledge, none of these tools have achieved a comprehensive, predictive framework that can accommodate outliers with atypical responses and reliably anticipate who will benefit most from vaccination.

Achieving this predictive capability is essential for personalized vaccination strategies. Identifying immune markers that predict response heterogeneity can guide tailored interventions to enhance vaccine effectiveness, especially in high-risk populations (10). This is crucial for rapid deployment of immunizations against pathogens with pandemic potential (19, 20).

To meet these challenges, we developed Immunaut, an open-source, data-driven framework for systems vaccinologists to unravel complex immune responses and predict vaccine outcomes. Through advanced modeling, Immunaut integrates multidimensional immune features to classify individuals into distinct immunophenotypic responder profiles, revealing how baseline characteristics shape vaccine responses. Applied here to a comprehensive LAIV datasets, Immunaut delineated responder groups with systemic humoral, mucosal, or T cell–mediated biases and uncovered critical biomarkers associated with effective LAIV responses. Beyond this specific application, Immunaut is readily adaptable to other vaccines and datasets, offering a robust foundation for precision vaccinology.

## Results

### Comprehensive immunoprofiling of LAIV responses reveals distinct immunophenotypic groups

**Figure 1:**
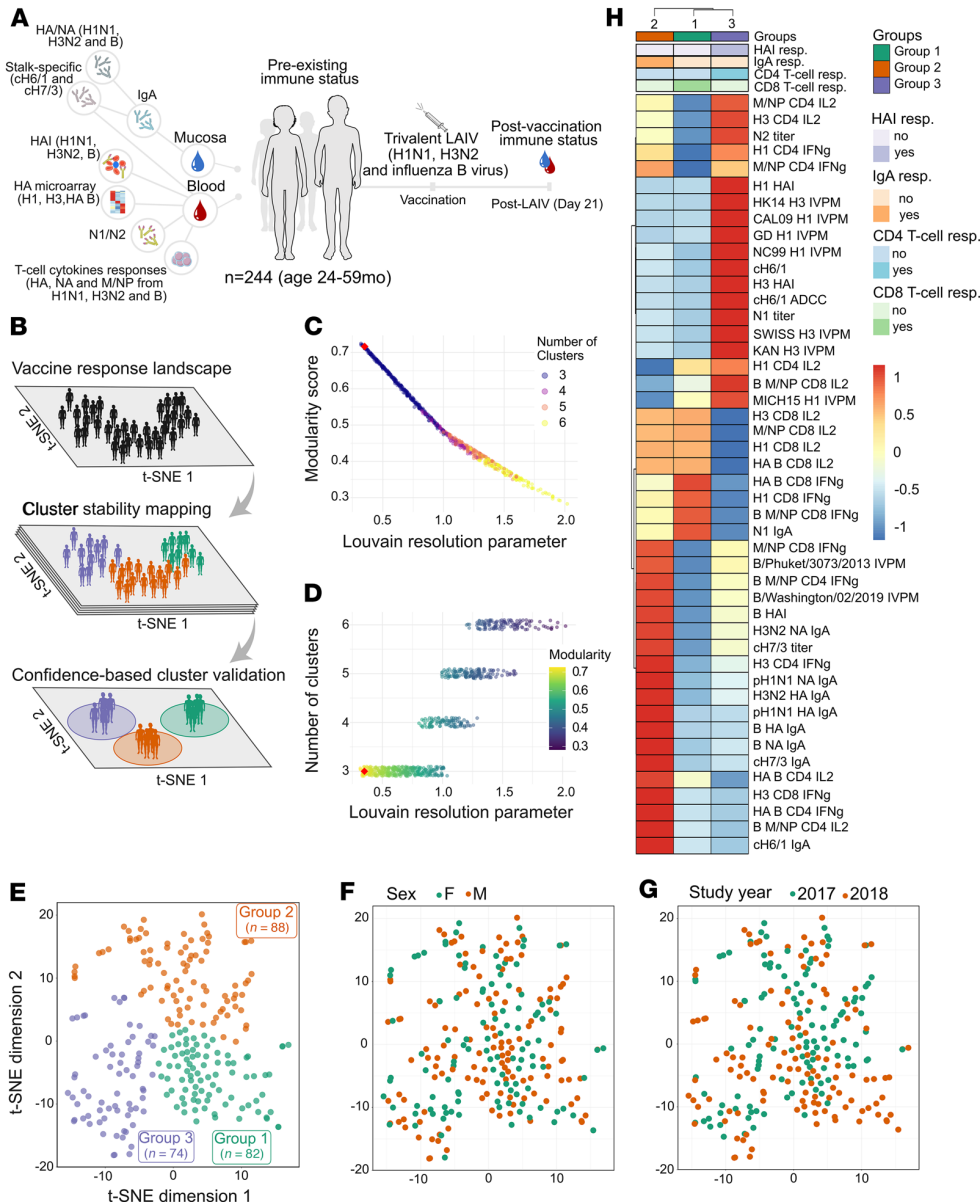
Immune response landscape mapping of LAIV reveals distinct immunophenotypic groups. (**A**) Cohort overview depicting all features used for unsupervised machine learning analysis: 244 children (24–59 months of age) vaccinated with LAIV; mucosal and blood samples collected on day 0 (prevaccination) and day 21 (postvaccination). Vaccine-induced immune responses calculated as fold-change relative to prevaccination levels. (**B**) Workflow schematic for automated clustering pipeline applying *t*-SNE dimensionality reduction, KNN graph construction, and Louvain community detection to identify distinct immunophenotypic clusters. (**C** and **D**) Louvain resolution sweep results used to assess cluster stability and select optimal number of clusters. (**C**) Modularity score plotted against Louvain resolution parameter, colored by number of clusters identified (3–6). High modularity indicates well-separated clusters. Red diamond indicates selected clustering parameters. (**D**) Number of clusters identified plotted against Louvain resolution parameter, colored by modularity score. Stability of 3-cluster solution (red diamond) is observed across range where modularity is maximal (Q ≈ 0.717). (**E**) Clustered *t*-SNE plot of fold-change data (post/pre-LAIV) revealing 3 distinct LAIV response phenotypes: group 1 (green, *n* = 82), group 2 (orange, *n* = 88), and group 3 (purple, *n* = 74). (*t*-SNE parameters: perplexity: 30; exaggeration factor: 4; max iterations: 10,000; theta: 0; eta: 500; K: 60 for KNN graph; final silhouette score: 0.40). (**F** and **G**) Clustering patterns overlaid with demographic factors on *t*-SNE map. (**F**) Clustering by sex (female, green; male, orange). (**G**) Clustering by study year (2017, green; 2018, orange). (**H**) Heatmap and hierarchical clustering display fold-change data for key immune features across 3 clusters (columns: groups 2, 1, and 3 from left to right). Rows represent immune features, clustered using Euclidean distance and Ward’s D2 method. Heatmap cells are colored based on scaled FC values from –1 (blue, low FC) to 1 (red, high FC). The top color bar indicates responder groups (group 1, green; group 2, orange; group 3, purple). Side color bars indicate qualitative response classifications derived from assays: HAI (purple: high, dark; low, light), IgA (orange: high, dark; low, light), CD4^+^ T cell (blue: high, dark; low, light), and CD8^+^ T cell (green: high, dark; low, light). Column cluster ordering optimized for visual clarity.

To define responder status to LAIV in 244 Gambian children (7), we focused on adaptive immune markers with paired baseline (day 0) and postvaccination (day 21) measurements, expressed as fold-change values (V21/V0; see Methods) (Figure 1A). Using fold-change accounted for interindividual variability in baseline immunity, capturing genuine vaccine-induced changes. We evaluated a comprehensive panel of antibody-mediated responses, including hemagglutination inhibition (HAI) titers, an indicator of antibodies that block the binding of the influenza virus to host cells (21). We used an influenza virus protein microarray to assess the breadth of antibody responses (22). This high-throughput platform profiles binding antibody responses across multiple influenza strains, including hemagglutinin (HA) proteins from various influenza A and B viruses. This allowed quantitative evaluation of serum antibody binding profiles before and after LAIV administration, providing insights into the specificity, magnitude, and breadth of the antibody responses, including cross-reactive responses. We also examined stalk-specific responses targeting conserved regions of the HA protein, including antibody-dependent cellular cytotoxicity activity measured against chimeric HA stalk constructs (e.g., cH6/1 and cH7/3) to assess cross-reactive immunity (23). Neuraminidase (NA) titers in blood and nasal mucosa offered insights into cross-protective responses (24). Complementing antibody profiles, we assessed T cell IFN-γ and IL-2 production upon stimulation with vaccine strain components (HA, NA, and matrix/nucleoprotein) to capture systemic cellular responses. Collectively, this panel of immunological assays provided a highly granular view of the magnitude and quality of immune responses elicited by LAIV administration, allowing us to capture a detailed immunophenotypic landscape.

This integrated, multimodal dataset served as input for the Immunaut machine learning framework (see Methods). To visualize patterns, we projected the high-dimensional data into a 2D space using *t*-distributed stochastic neighbor embedding (*t*-SNE) (Figure 1B). We then constructed a K-nearest neighbors (KNN) graph based on Euclidean distances in this reduced space. We applied the Louvain community detection algorithm to identify distinct immunophenotypic groups, which partitions the graph to maximize the modularity score (Q), a measure of clustering quality where a higher modularity score indicates more distinct and well-separated clusters. We systematically evaluated clustering stability by applying the algorithm across a range of resolution values (*r*), where lower resolutions yield fewer, larger clusters and higher resolutions produce more, smaller ones. This assessment revealed a resolution range where modularity reached a high and stable plateau (Q ≈ 0.717, Figure 1C), signifying a robust and well-defined community structure, and the number of clusters consistently converged to 3 (Figure 1D). This provides quantitative evidence that this partitioning reflects distinct biological subtypes rather than arbitrary divisions sensitive to parameter tuning.

The final 3-cluster partition is visualized on the *t*-SNE projection (Figure 1E), comprising group 1 (green, *n* = 82), group 2 (orange, *n* = 88), and group 3 (purple, *n* = 74). The average silhouette score of 0.4 indicates moderately distinct clusters. We observed no substantial enrichment of specific sexes (Figure 1F) or study years (Figure 1G) within any cluster, suggesting the clustering captures genuine immunophenotypic differences independent of these external biases known to affect immune responses to vaccines (25–27).

**Figure 2:**
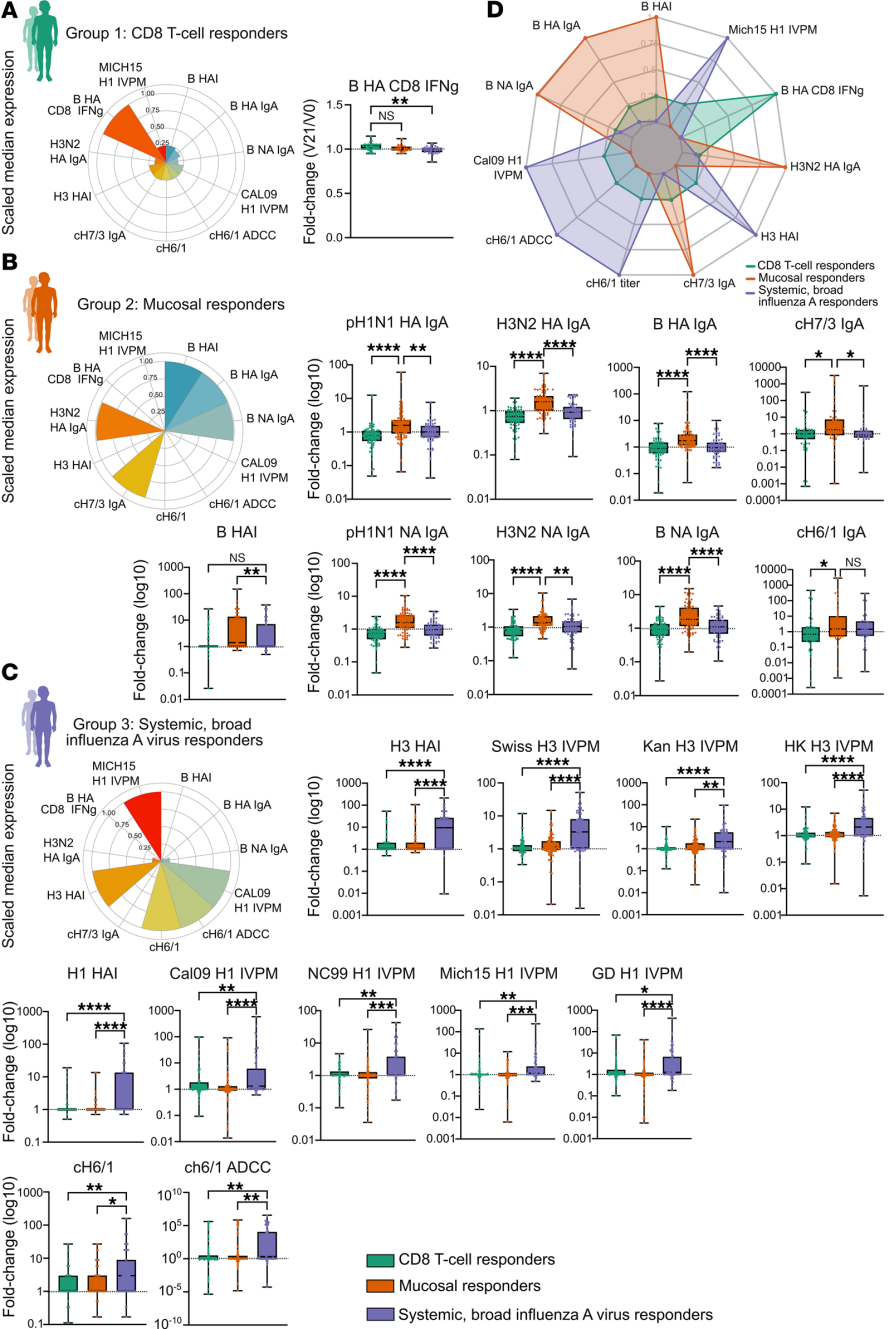
Vaccine response immune signatures defining LAIV responder types. (**A**) Polar plot summarizing scaled median expression of key immune features in CD8^+^ T cell responders (group 1, green). CD8^+^ T cell responders are characterized by robust influenza B virus HA–specific CD8^+^ IFN-γ responses and limited humoral immunity, with median feature values represented in the polar plot and fold-change comparisons shown in the adjacent box plot. (**B**) Polar plot for mucosal responders (group 2, orange) illustrating strong mucosal IgA responses, particularly stalk-specific (cH7/3 IgA) and H3N2 virus HA–specific IgA antibodies and influenza B virus–specific responses. Box plots detail fold changes (shown as log_10_) for various immune features, highlighting systemic (influenza B virus HAI) and mucosal immune activation (IgA). (**C**) Polar plot depicting systemic, broad influenza A virus responders (group 3, purple), showing elevated systemic antibody responses to multiple influenza A virus strains (e.g., H1, H3), as well as cross-reactive IgG and antibody-dependent cellular cytotoxicity activity. Box plots show fold-change values (log_10_) for each immune marker across responder groups. (**D**) Integrated radar plot comparing scaled median immune expression profiles across all responder groups (CD8^+^ T cell responders in green, mucosal responders in orange, systemic broad influenza A virus responders in purple), emphasizing distinct immune feature distributions. This integrative visualization highlights the unique baseline and postvaccination immune landscapes that define each responder profile. Box plots denote minimum to maximum values, and points are all individuals within the group. *P < 0.05, **P < 0.01, ***P < 0.001, and ****P < 0.0001, by 1-way ANOVA Kruskal-Wallis test with Dunn’s multiple-comparison test to adjust for multiple testing.

Individuals in group 1 (*n* = 82) displayed a distinct profile characterized by CD8^+^ T cell–mediated responses and notably low CD4^+^ T cell IFN-γ activity (Figure 1H). This group showed elevated IFN-γ and IL-2 production by CD8^+^ T cells upon stimulation, with the most pronounced responses against influenza B virus HA and matrix/nucleoprotein antigens (Figure 1H). Influenza B virus–specific CD8^+^ T cell IFN-γ responses were statistically significant compared with group 3 (Figure 2A). Conversely, humoral and IgA responses in group 1 were minimal or absent (Figure 1H and Figure 2, A and D). Although some N1-specific IgA responses were detected (Figure 1H), these responses were not statistically significant and were comparable to those observed in group 2, indicating that the N1 IgA responses were not a distinguishing feature of group 1 (Supplemental Figure 1A; supplemental material available online with this article; https://doi.org/10.1172/JCI189300DS1). Based on these results, we termed individuals in group 1 as “CD8^+^ T cell responders.”

In contrast, group 2 (*n* = 88) individuals exhibited a profile dominated by mucosal IgA responses (Figure 1H). This group showed statistically significant induction of mucosal IgA antibodies across all antigens and strains tested, including against chimeric HA stalk constructs, indicating the induction of antibodies targeting conserved HA regions (Figure 2B). The consistent IgA increase was unique to group 2 and not observed in groups 1 or 3 (Figure 2, B and D), validating their classification as mucosal responders. Group 2 also exhibited significant seroconversion to influenza B viruses (Figure 1H), evidenced by substantial increases in HAI titers (Figure 2B). Although antibody binding responses to influenza B viruses measured by influenza virus protein microarray were elevated (Figure 1H), they were not statistically significant (Supplemental Figure 1B). This suggests humoral immunity in group 2 included both mucosal and systemic antibody responses against influenza B virus.

Group 3 (*n* = 74) individuals showed robust systemic antibody responses to influenza A viruses (Figure 1H). This was evidenced by significant increases in HAI titers for both H1N1 and H3N2 strains (Figure 2C). The antibody responses demonstrated breadth, with substantial increases in binding to HA subtypes from multiple contemporary and historical H1N1 and H3N2 strains not present in the vaccine (Figure 2C). Elevated responses were also observed against the cH6/1 chimeric HA construct, including increased antibody-dependent cellular cytotoxicity (Figure 2C). Significantly higher N1 titers were also detected (Supplemental Figure 1C), supporting a coordinated response targeting conserved epitopes. Although some CD4^+^ T cell responses were elevated (Supplemental Figure 1C), significant IgA responses were absent, indicating predominantly systemic immunity (Figure 2, B and D). This finding supports classifying them as systemic broad influenza A virus responders.

### Predictive modeling of LAIV response phenotypes based on baseline immune profiles

**Figure 3:**
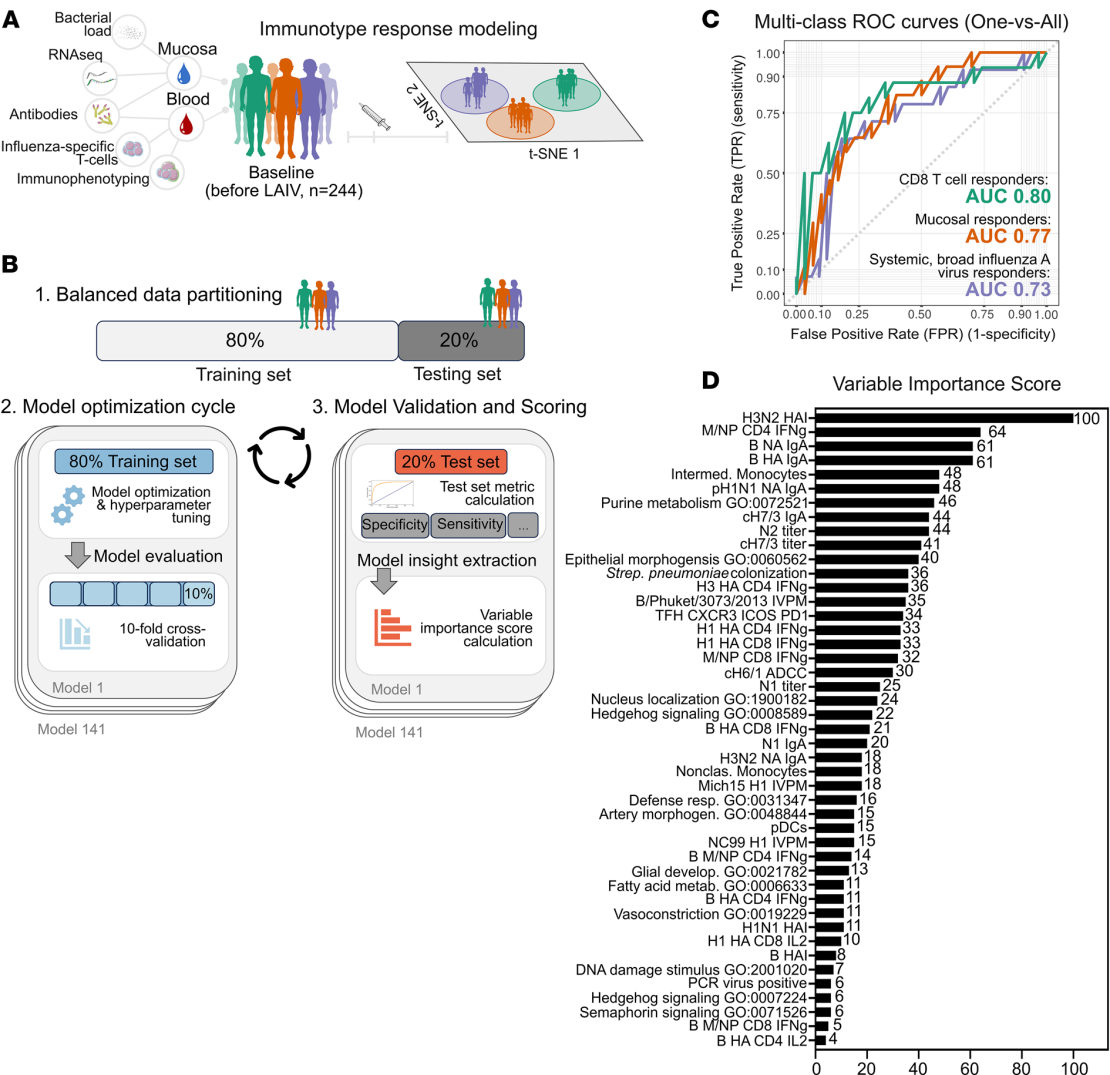
Automated machine learning framework for mapping and predicting LAIV immunogenicity response phenotypes. (**A**) Overview of the automated machine learning framework developed to predict LAIV response phenotypes using baseline immune data from mucosal and blood samples, capturing multidimensional immune parameters such as transcriptomics, antibody titers, bacterial load, flu-specific T cell responses, and comprehensive immunophenotyping. (**B**) Step 1, balanced data partitioning: the dataset is split into training (80%) and testing (20%) sets, ensuring proportional representation of each immunophenotypic group (CD8^+^ T cell; mucosal; and systemic, broad influenza A responders) to maintain predictive accuracy across classes. Step 2, model optimization cycle: 10-fold cross-validation and hyperparameter tuning are applied across 141 machine learning models, each iteratively trained and validated to identify the best predictors of vaccine response. Step 3, model evaluation and scoring: predictive performance metrics, including specificity, sensitivity, and AUC, are calculated on the test set (20%) for model validation. Feature importance scores are computed for each baseline variable, providing a ranked analysis of each immune parameter’s contribution to LAIV response prediction. (**C**) Multiclass ROC plot of the gradient boosting machine model evaluated on the test set (20%), displaying predictive accuracy across all 3 classes: CD8^+^ T cell responders (green); mucosal responders (orange); and systemic, broad influenza A responders (purple) in a one-versus-all comparison. (**D**) Variable importance score table for the gradient boosting machine model, showcasing the cumulative importance of the selected baseline features across the 3 predicted classes, highlighting the most influential parameters in LAIV immunogenicity prediction.

We next sought to determine whether prevaccination immune profiles could predict an individual’s response type. To achieve this, we used comprehensive baseline immunological measurements before vaccination (Figure 3A), including antibody profiles, T cell responses, *Streptococcus* *pneumoniae* load (pneumococcal carriage density), asymptomatic respiratory viral presence, RNA pathway scores from nasal samples, and frequencies of various immune cell subsets such as monocytes, plasmacytoid and myeloid DCs (pDCs and mDCs), and T follicular helper (Tfh) cells (28) relevant to LAIV, which relies on both innate and adaptive immune pathways to induce protection (5, 6, 29, 30).

To model the mapped vaccine responses, we applied the Sequential Iterative Modeling OverNight (SIMON) platform, which is designed for high-dimensional datasets with substantial interindividual variability (17, 31) (Figure 3B). We systematically tested 141 machine learning algorithms to ensure the selection of the most accurate and biologically meaningful model (17, 31–33). We employed 10-fold cross-validation during model training to enhance robustness and mitigate overfitting, and we assessed performance on a held-out test set to ensure generalizability. Out of the 141 models tested, 26 achieved a test set area under the receiver operating characteristic curve (AUC) above 0.7, underscoring the predictive strength of our baseline immune profiles (Supplemental Table 1).

Among all models, the gradient boosting machine model was the top performer (Supplemental Table 1). It achieved an accuracy of 59.57% (exceeding the null accuracy of 36.17%; *P* = 0.0009), a balanced accuracy of 71.67%, an F1-score of 0.6286, a precision of 0.6902, and a recall of 0.6471, highlighting its capacity to balance false-positives and false-negatives and an overall AUC of 0.8. One-versus-all AUCs confirmed robust performance across individual classes: 0.80 for CD8^+^ T cell responders, 0.77 for mucosal responders, and 0.73 for systemic broad influenza A virus responders (Figure 3C). Training gradient boosting machine models on individual or pairwise data types showed that removing any primary data modality reduced performance, demonstrating that integration of diverse features was essential for high accuracy (Supplemental Table 2). The gradient boosting machine model’s capacity for feature importance estimation, its ability to manage high-dimensional data, and its robustness to missing data (Supplemental Figure 2) make it a powerful tool for this classification task.

Next, we identified the baseline features that were most critical for classification (Figure 3D). The top predictor was the baseline HAI geometric mean titer against H3N2 (score 100), indicating that preexisting systemic immunity drives the response type. However, high baseline mucosal IgA against various influenza antigens, including influenza B/Victoria/2/87-like lineage HA and NA (61), pH1N1 HA (48), N1 (20), H3N2 NA (18), and cH7/3 IgA (44) was also pivotal, underscoring the complementary roles of systemic and mucosal immunity. Key cellular parameters, such as IFN-γ–producing T cells (e.g., influenza A virus matrix/nucleoprotein CD4 IFN-γ, score 64; H1N1 and H3N2 HA CD4 IFN-γ, 33 and 36; influenza B HA CD8^+^ IFN-γ, 21) and Tfh cell frequencies (34), also surfaced as prominent predictors. Additionally, innate immune cells, pneumococcal carriage density (36), and asymptomatic respiratory viral infection (6) emerged as critical modulating factors. Notably, baseline nasal RNA-derived Gene Ontology (GO) pathways, encompassing metabolism (GO:0072521, variable importance score 46), morphogenesis (GO:0060562, score 40), and Hedgehog signaling (GO:0008589, GO:0007224, scores 22 and 6), contributed substantially, pointing to a context-dependent model of vaccine responsiveness where tissue-level processes shape the immune response.

Collectively, these observations suggest that LAIV response phenotypes arise not from a single dominant factor but emerge from a finely tuned network of systemic and local immunity, innate and adaptive cellular components, and underlying tissue-level processes.

### Identifying preexisting immune landscapes that shape LAIV responses

**Figure 4:**
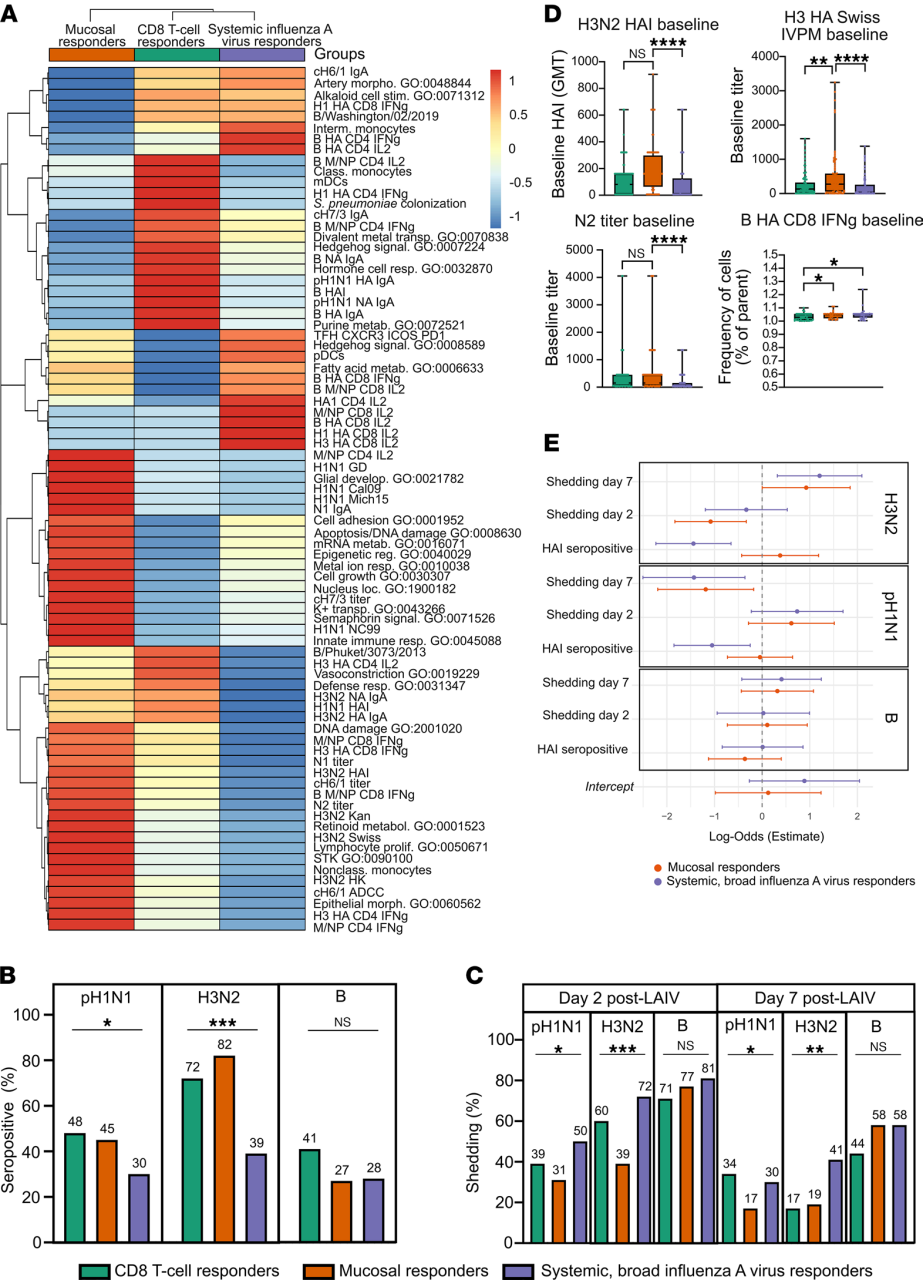
Baseline immune landscape and viral shedding profiles predictive of LAIV response groups. (**A**) Heatmap of baseline immune features predictive of LAIV response groups, organized by hierarchical clustering to show feature relationships and variations across groups (Euclidean distance, Ward’s D2 clustering method). Each cell reflects a scaled expression level, with red representing high expression and blue indicating low expression, revealing the distribution of immune features at baseline across the identified immunophenotypic clusters. (**B**) The proportion of seropositive children (HAI titer ≥10) at baseline (before vaccination) within each responder group and across all 3 LAIV-strains, pH1N1, H3N2, and influenza B virus. (**C**) The proportion of children that shed LAIV strains (pH1N1, H3N2, and B) on day 2 and day 7 after vaccination across all 3 responder groups. (**D**) Box plots showing baseline features, including H3N2 HAI geometric mean titer (gmt), titer of antibodies binding H3 HA from A/Switzerland/9715293/2013 analyzed by influenza virus protein microarray (H3 HA SWISS IVPM), titer of antibodies binding NA from group 2 (N2) and frequency of influenza B virus HA–specific CD8^+^ T cells producing IFN-γ across all 3 responder groups, CD8^+^ T cell responders (green); mucosal responders (orange); and systemic, broad influenza A virus responders (purple). Box plots denote minimum to maximum values, and points are all individuals within the group. *P < 0.05, **P < 0.01, ***P < 0.001, and ****P < 0.0001, by 1-way ANOVA Kruskal-Wallis test with Dunn’s multiple-comparison test to adjust for multiple testing. (**E**) Forest plots showing log-odds estimates from a logistic regression model. The plots illustrate the association of the mucosal responder (orange) and systemic, broad influenza A virus responder (purple) groups with the outcomes of viral shedding (day 2 and 7) and HAI seropositivity, relative to the CD8 T-cell responder group which serves as the reference category. The analysis is stratified by LAIV strain (H3N2, pH1N1, and B), and the error bars represent the confidence intervals for the log-odds estimates.

To delineate the preexisting immune landscapes that define each group, we hypothesized that specific baseline conditions characterize each responder class. To test this, we combined machine learning–derived insights with exploratory analyses of baseline seropositivity, viral shedding, and detailed immunological profiling. The resulting patterns suggest that historical exposure to influenza strains plays a pivotal role in shaping the immune response to LAIV (Figure 4).

Children who became CD8^+^ T cell responders had a distinctive baseline signature (Figure 4A). Before vaccination, this group had significantly higher seropositivity for H1N1 (48%) and H3N2 (72%) (*P* = 0.049 and *P* < 0.0001) and elevated baseline HAI responses (Figure 4, A and B). They also had elevated baseline levels of influenza virus–specific IgA in nasal secretions, targeting multiple LAIV strains and cH7/3 chimeric stalk construct, features identified by the machine learning model as important (Figure 4A). After vaccination, this group showed significantly reduced shedding of H3N2 by day 7 (17% shedding rate), an association confirmed by logistic regression (β = 1.21, *P* = 0.0078) (Figure 4E). Baseline nasal transcriptional analysis identified enrichment of pathways, including purine metabolism (GO:0072521; score 46) and regulation of defense response (GO:0031347; score 16) (Figure 4A). These children also had elevated baseline *S*. *pneumoniae* loads (score 36), Hedgehog signaling pathway (GO:0007224, score 6), and asymptomatic respiratory viruses (predominantly rhinovirus) detected before vaccination (χ_2_ test *P* = 0.57; Supplemental Figure 3). The frequency of circulating classical monocytes and mDCs was also elevated at baseline (Figure 4A).

Mucosal responders showed high baseline seropositivity to influenza A viruses (H3N2 = 82%, H1N1 = 45%) but a lower rate against the influenza B virus (27%) (Figure 4, A and D). High baseline H3N2 HAI titers were prominent, and reactivity extended to multiple H1N1 strains (e.g., influenza virus protein microarray assay, N1, N2, cH6/1, cH7/3), indicating extensive prior exposures (Figure 4, A and D). Nasal epithelial signatures related to morphogenesis (GO:0060562), innate immunity (GO:0045088), mRNA metabolism (GO:0016071), epigenetic regulation (GO:0040029), retinoid metabolism (GO:0001523), and lymphocyte proliferation (GO:0050671) pointed toward a state of mucosal readiness. This preexisting influenza A virus immunity allowed for efficient containment of LAIV’s influenza A virus strains, with shedding of H1N1 and H3N2 strains significantly reduced by day 2 (*P* = 0.043 and *P* < 0.0001) and minimal shedding observed by day 7 (Figure 4C). Regression analysis confirmed this association with lower shedding of H3N2 at day 2 (β = –1.08, *P* = 0.0047) and H1N1 at day 7 (β = –1.18, *P* = 0.021) (Figure 4E). In contrast, shedding of influenza B virus persisted longer, and as per their definition, these children seroconverted to the influenza B virus after vaccination.

Systemic, broad influenza A virus responders had significantly lower prevaccine seropositivity for H1N1 (30%) and H3N2 (39%) (*P* = 0.049 and *P* < 0.0001, respectively; Figure 4B), further confirmed by the regression analysis (low baseline H1N1 (β = –1.05, *P* = 0.010) and H3N2 seropositivity (β = –1.44, *P* < 0.001) (Figure 4E). Regression analysis confirmed that systemic responder status was significantly associated with low baseline H1N1 (β = –1.05, *P* = 0.010) and H3N2 seropositivity (β = –1.44, *P <* 0.001), and with persistent H3N2 shedding at day 7 (β = 1.21, *P* = 0.0078) (Figure 4E). At baseline, these responders had a higher frequency of circulating intermediate monocytes, pDCs (score 15), and Tfh cells (score 34) that facilitated a robust systemic response (Figure 4A). They also displayed a rich array of T cell functional responses to multiple influenza antigens (Figure 4, A and D).

Taken together, these findings show how limited baseline immunity can lead to stronger systemic responses, while a pre-primed environment streamlines early containment and clears the path for novel responses.

### The integrative machine learning interpretation approach reveals determinants of LAIV response profiles and predictors of immunogenicity

Interpreting multiclass machine learning models is challenging due to the complex interplay between features across outcome classes. Features selected by the model can represent both enriched and reduced characteristics across groups, complicating direct interpretation. We adopted a multifaceted framework to extract mechanistic insights, integrating pathway-level analysis, group-specific feature impact evaluation, and hierarchical examination of feature splits in the model’s decision structure.

First, baseline features were mapped onto predefined biological pathways or functional categories (Supplemental Table 3). Immune metrics were assigned to humoral, cellular, or mucosal immunity compartments, with separate groupings for microbial load, antigen presenting cell (APC) populations, and Tfh cells. GO terms from nasal transcriptomic data were grouped into broader categories, such as metabolic and epigenetic regulation, epithelial barrier integrity and tissue remodeling, immune and inflammatory regulation, and others.

**Figure 5:**
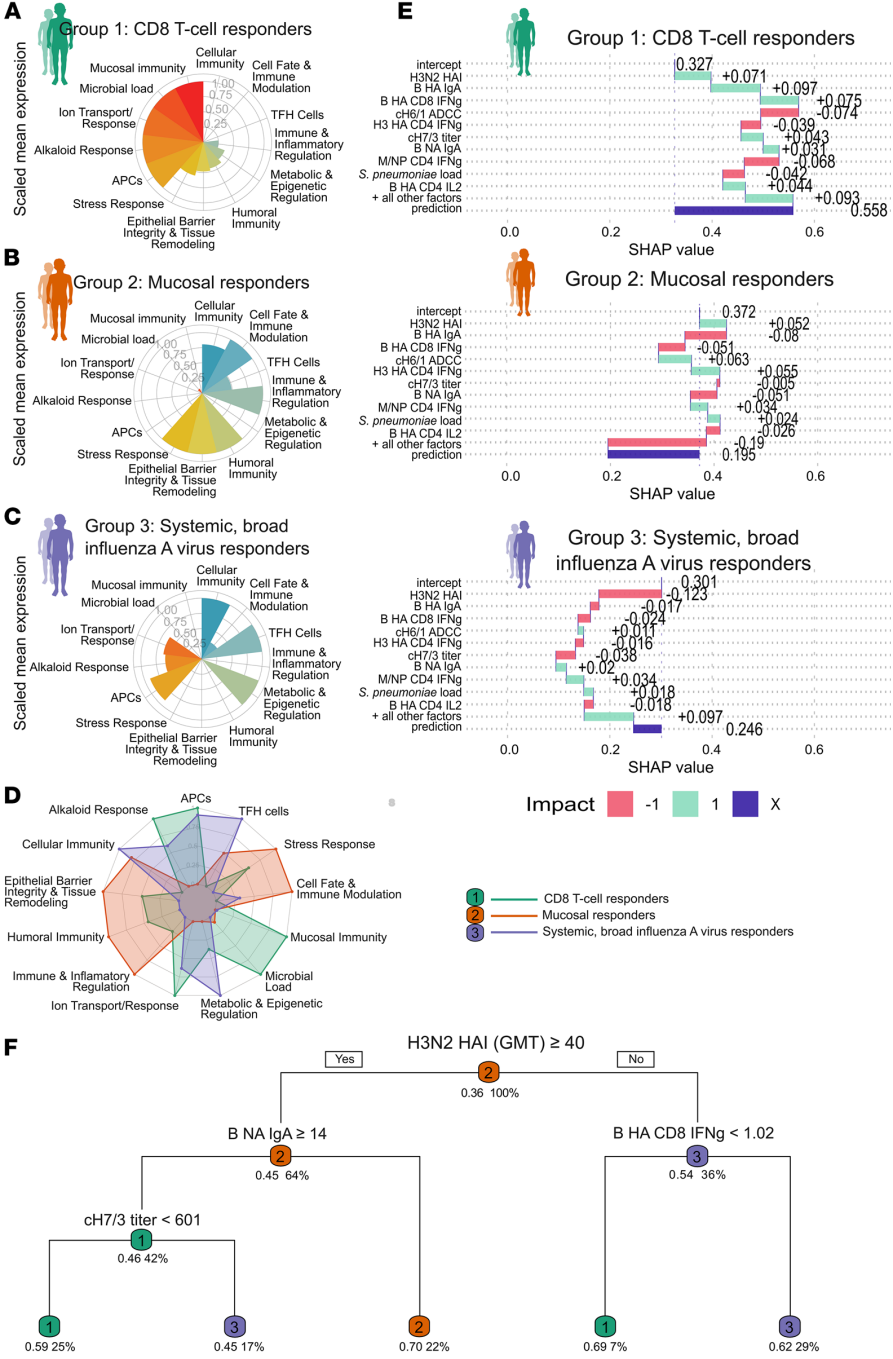
Baseline immune features and pathway-level determinants of LAIV responder profiles. (**A**–**C**) Polar plots illustrating scaled median expression of immune pathways across 3 responder groups: (**A**) CD8^+^ T cell responders (group 1, green); (**B**) mucosal responders (group 2, orange); and (**C**) systemic, broad influenza A virus responders (group 3, purple). (**D**) Combined radar plot showing integrated immune pathway signatures across the 3 responder groups, highlighting intergroup differences in pathway activation. (**E**) SHAP (SHapley Additive exPlanations) summary plots showing the contribution of baseline features to model predictions for each responder group (CD8^+^ T cell responders, group 1, green; mucosal responders, group 2, orange; and systemic, broad influenza A virus responders, group 3, purple). The intercept represents the baseline prediction before feature contributions. All other factors include the combined effect of features not displayed in the top 10 contributors. Prediction (purple bar) is the final probability derived by summing the intercept, top 10 feature contributions, and all other factors. Feature impacts are color coded as follows: green (positive, 1) increases the likelihood of belonging to the group, and red (negative, –1) decreases it. The top 10 features are ranked by their contribution to the prediction, providing insights into key drivers of LAIV response profiles. (**F**) The decision tree depicts the splits made at each node based on immune feature thresholds. Splits are chosen to maximize class separation, with fitted class probabilities displayed as group 1 (CD8^+^ T cell responders, green), group 2 (mucosal responder, orange), and group 3 (systemic, broad influenza A virus responders, purple) for each terminal node. The coverage percentage represents the proportion of observations falling under each rule. Nodes are labeled with thresholds and the conditions that define group separation, with terminal nodes representing the predicted group and associated probabilities.

Pathway-level scores revealed distinct baseline signatures for each group (Figure 5, A–D). The dominant signature for CD8^+^ T cell responders included mucosal immunity and microbial load pathways (Figure 5, A and D). For mucosal responders, key baseline pathways encompassed humoral immunity, immune regulation, epithelial barrier integrity, immune modulation, and stress response (Figure 5, B and D). For systemic, broad influenza A responses, the baseline signature was marked by enriched cellular immunity, APC function, and Tfh cell support (Figure 5, C and D).

To gain more granular insights, we used SHAP (SHapley Additive exPlanations) analysis to quantify each predictor’s observation-specific contribution (34) (Figure 5E). For CD8^+^ T cell responders, elevated H3N2 HAI titers and influenza B virus-directed mucosal and cellular immunity positively shifted probabilities (Figure 5E). In mucosal responders, influenza B virus NA IgA was a key local contributor (Figure 5E). For systemic, broad influenza A virus responders, SHAP revealed how lower preexisting H3N2 immunity and supportive Tfh-APC-metabolic landscapes promoted broader antibody repertoires (Figure 5E).

Finally, decision-tree analysis established quantitative thresholds for key features distinguishing the groups (Figure 5F). Baseline H3N2 HAI was the primary discriminator, with a threshold of 40. Subsequent splits identified specific feature combinations associated with different responder likelihoods. Children exceeding this H3N2 HAI threshold (>40) combined with moderate levels of B/Victoria/2/87-like lineage NA IgA (titer ≥14) but lower cH7/3 titers (<601) had a 59% likelihood of developing CD8^+^ T cell responses. Alternatively, a similarly high H3N2 baseline (≥40) combined with low B/Victoria/2/87-like lineage NA IgA (<14) favored the mucosal responder type (70% likelihood). Low H3N2 HAI (<40) combined with higher baseline influenza B virus HA–specific CD8^+^ T cell IFN-γ responses associated with a 62% likelihood of the systemic broad influenza A response profile. These findings provide quantitative thresholds demonstrating how specific combinations of baseline features relate to distinct LAIV-induced immunophenotypes.

## Discussion

In this study, we introduced Immunaut, an integrative machine learning approach, to decipher how the preexisting immune landscape in children dictates outcomes after LAIV administration. Moving beyond traditional analyses, often limited to linear models and single biomarkers, our methodology synthesized high-dimensional prevaccination data to provide a cohesive, systems-level view of LAIV immunogenicity. This approach identified 3 distinct postvaccination immunophenotypes: CD8^+^ T cell responders; mucosal (and influenza B humoral) responders; and systemic, broad influenza A responders, each linked to specific baseline signatures.

A key strength of our strategy is its ability to reveal nuanced biological states by capturing nonlinear interactions. By employing multiple modeling techniques, Immunaut translated complex profiles into interpretable biological insights, allowing us to understand how combinations of factors shape the ultimate response. For example, identifying the mucosal responders highlights this advantage; standard analyses focused on systemic HAI antibodies might misclassify these individuals as poor responders, overlooking substantial mucosal immunity. Although the achieved predictive accuracy (AUC 0.80) is statistically significant and highlights the potential of baseline immunophenotyping, further improvements are needed for direct clinical application. The model’s primary utility is its power to integrate complex datasets, identify responder subgroups, and pinpoint key baseline features driving response heterogeneity — crucial steps in advancing our understanding toward personalized vaccinology.

CD8^+^ T cell responders started with a baseline reflecting extensive prior influenza exposure and heightened mucosal readiness. They exhibited higher baseline seropositivity for H1N1 and H3N2, elevated HAI responses, and robust levels of influenza virus–specific IgA in the nasal mucosa. This potent preexisting antibody profile contributes to rapid viral containment, evidenced by reduced viral shedding. Their baseline nasal transcriptome showed enrichment in defense and metabolism pathways, and they had higher *S*. *pneumoniae* loads and frequencies of classical monocytes and mDCs, suggesting a state of immune vigilance (8, 35, 36). We propose that this combination of strong preexisting immunity and a primed innate environment leads to efficient early control of LAIV, limiting the antigenic stimulus and favoring the recall of memory CD8^+^ T cells, resulting in the observed T cell–dominant phenotype.

Conversely, the mucosal responders emerged from a different immunological starting point, with high baseline seropositivity to influenza A viruses but substantially lower immunity against influenza B. Their nasal transcriptome indicated a well-regulated mucosal interface. We interpret this as the strong preexisting influenza A immunity facilitating rapid control of the LAIV A strains, as evidenced by the participants’ significantly reduced H1N1 and H3N2 shedding soon after vaccination. However, the initial encounter with influenza A viral strains at the mucosal surface appears sufficient to trigger a local immune response, likely involving recall of existing mucosal memory B cells. Concurrently, the lower baseline immunity against influenza B virus allows more persistent replication of this component within the nasal mucosa, providing a sustained antigenic stimulus. We propose that this sustained local activation, driven by the persistent influenza B component, provides the necessary help for a robust de novo response leading to influenza B seroconversion and boosts the recall IgA response against the influenza A components at the mucosa. The baseline enrichment in epithelial and regulatory pathways suggests a mucosal environment capable of orchestrating this complex, temporally staggered, yet ultimately broad local antibody response. This configuration results in the defining phenotype: rapid systemic containment of influenza A, seroconversion to influenza B, and robust mucosal IgA responses to all 3 LAIV strains.

The systemic, broad influenza A virus responder phenotype was associated with lower prevaccine seropositivity to influenza A strains, permitting higher initial viral replication and a strong antigenic stimulus. Decision-tree analysis revealed 2 distinct pathways to this outcome. One involved individuals with preexisting immunity across multiple influenza A domains (high baseline H3N2 HAI, high B/Victoria NA IgA and cH7/3), suggesting a response dominated by the recall of preexisting cross-reactive memory B cells, a mechanism consistent with phenomena like original antigenic sin or back-boosting (37, 38), effectively leveraging prior exposures to generate breadth (39, 40). The second pathway occurred in naive individuals (low prevaccination level of H3N2 HAI) with increased frequencies of T cell, APC, and Tfh signatures. Here, the breadth appears driven by T cells, which help facilitate de novo B cell activation, potentially involving cross-reactive T cells (41), likely fueling the germinal center activity (41, 42). Our findings identified 2 distinct routes to achieving broad systemic influenza A immunity after LAIV administration: one relying on recalling antibody memory, and the other leveraging cellular support to build breadth from a more naive state.

Although demonstrated for LAIV, the Immunaut framework is generalizable and can be applied to other vaccines or infections to accelerate biomarker discovery and rational vaccine design. Limitations remain, including the need for validation in larger, more diverse cohorts across different ages, genetic backgrounds, and geographical locations to confirm the generalizability of these signatures. Also, targeted mechanistic experiments are needed to establish causality for the proposed pathways. Future work incorporating expanded multiomics datasets will further refine our understanding and predictive capabilities.

In summary, this study leverages an integrative machine learning approach, Immunaut, to provide a high-resolution map of how preexisting immune landscapes dictate LAIV outcomes in children. By identifying distinct prevaccination signatures and quantitative thresholds that predict divergent response trajectories, our work offers crucial insights into the complex interplay governing vaccine immunogenicity. This represents a substantial step toward precision vaccinology, providing a framework for understanding and predicting vaccine responses.

## Methods

### Sex as a biological variable

This study included 244 children of both sexes, aged 24–59 months. Sex was considered as a demographic variable.

### Study participants

We compiled data from multiple research projects that evaluated immune responses to a trivalent LAIV (Nasovac-S, based on the A/Leningrad/134/17/57 master donor strain, which is in use in Russia) among children in The Gambia. The cohort comprised 244 children aged 24–59 months who received the LAIV during 2017 and 2018 as part of an open-label, prospective, observational, phase 4 immunogenicity study nested within a larger randomized trial (ClinicalTrials.gov NCT02972957) (7). Eligible participants were healthy children with no history of respiratory illness in the preceding 14 days and no prior influenza vaccination. Exclusion criteria included serious active medical conditions (e.g., chronic diseases, severe malnutrition, genetic disorders), known immunodeficiency, hypersensitivity to vaccine components, recent use of immunosuppressive therapies, and contraindications to LAIV administration. After community sensitization, recruitment was conducted in Sukuta, a periurban area in The Gambia. Participants recruited in 2017 (*n* = 118) received the 2016–2017 northern hemisphere formulation, which included strains A/17/California/2009/38 (H1N1)pdm09-like, A/17/Hong Kong/2014/8296 (H3N2)-like, and B/Texas/02/2013-like (B/Victoria/2/87-like lineage). In 2018 (*n* = 135), participants received the 2017–18 formulation in which the H1N1 component was updated to A/17/New York/15/5364 (H1N1)pdm09-like; the H3N2 and B strains remained unchanged. Nine individuals in the 2018 cohort withdrew or missed the final study visit, leaving 244 children for the final analyses (see ref. 7 for the study profile). Whole blood and serum were collected before vaccination (day 0) and on day 21 after vaccination. Nasopharyngeal swabs were collected at days 0, 2, and 7 after LAIV administration using flocked swabs (Copan FLOQSwabs) and stored in RNAprotect Cell Reagent (QIAGEN) for viral shedding assessment and microbiome analyses. To evaluate mucosal antibody responses, oral fluid samples were collected at days 0 and 21 after LAIV administration using Oracol Plus swabs (Malvern Medical Development). Whole blood samples were drawn for serum separation, flow cytometry, and transcriptomic analyses. All samples were stored at –70°C until processing.

### Datasets encompassing a wide array of immune parameters

#### Humoral immune responses.

HAI assays (21) were performed according to standard protocols on all 244 children using prevaccination (day 0) and postvaccination (day 21) samples using vaccine strain-matched antigens to assess seroconversion, defined as a 4-fold or greater increase in HAI titers to 1:40 or greater from day 0 to day 21 (7). This allowed for the evaluation of antibody responses against all 3 LAIV-vaccine strains: A(H1N1) pdm09, A(H3N2), and B/Victoria/2/87-like lineage influenza virus strains. Influenza virus–specific IgA in oral fluids was quantified using a protein microarray with recombinant HA and neuraminidase proteins and normalized to total IgA in the sample (measured by ELISA) (43). A 2-fold increase in the proportion of influenza virus–specific IgA was considered a significant mucosal antibody response. An influenza virus protein microarray was performed on 239 study participants with both time points to determine the cross-reactive binding of serum antibodies against a panel of HA proteins from various influenza virus strains, including both vaccine-matched, drifted, and historical variants (44). Antibody-dependent cellular cytotoxicity activity was assessed on all study participants using reporter cell lines expressing Fc gamma receptors in the presence of chimeric H6/1 HA protein (H6 head domain combined with an H1 stalk domain), measuring the ability of antibodies to bind to group 1 HA stalk and mediate effector cell functions, exactly as described previously (45). Briefly, we employed a stable Madin-Darby canine kidney (MDCK) cell line expressing a chimeric H6/1 HA, wherein the H6 head domain renders this target largely free of head-specific human antibodies, thereby enabling focused detection of stalk-directed responses. After seeding these cH6/1 MDCK cells in 96-well plates, serially diluted serum or monoclonal antibodies were added, followed by Jurkat effector cells engineered to express human FcγRIIIa (V158 variant). The assay was incubated for 6 hours, after which luminescence was measured as an indicator of effector cell activation via the Fc-HA interaction. ELISA based on standardized protocols was used to measure IgG levels to serum NA from N1 and N2, serum group 1 and 2 stalk-specific IgG using chimeric HA constructs (cH6/1 and cH7/3; H7 head domain on top of an H3 stalk domain), secretory IgA in oral secretions to N1 NA, and group 1 stalk in 242 study participants.

#### Cellular immune responses.

T cell responses before and on day 21 after LAIV administration were measured by stimulating fresh whole blood with overlapping 15–18-mer peptide pools covering vaccine-matched HA (H1, H3, and B/Victoria/2/87-like HA), nucleoprotein, and matrix proteins (219 study participants). Intracellular cytokine staining for IFN-γ and IL-2 was performed, and responses were analyzed using flow cytometry, as previously described (7).

#### Viral shedding, density of S. pneumoniae, and viral load.

Nasopharyngeal swabs from 244 participants were assessed for LAIV strain shedding on days 2 and 7 after LAIV administration using reverse-transcription PCR (RT-PCR) assays targeting HA genes as previously described (7). Quantitative RT-PCR provided viral load measurements expressed as log_10_ egg infectious dose equivalents per milliliter. Additionally, the presence and density of nasopharyngeal *S*. *pneumoniae* before vaccination were quantified as previously described (9). Baseline samples were tested for the presence of respiratory viruses using a multiplex real-time PCR method, as detailed in the original publication (28). The assay panel included influenza A and B viruses, respiratory syncytial virus types A and B, human parainfluenza viruses 1–4, human metapneumovirus, adenovirus, seasonal coronaviruses (229E, OC43, NL63), and human rhinovirus.

#### Immunophenotyping.

Multicolor flow cytometry panels were utilized to quantify frequencies of innate immune cell subsets before vaccination in 130 participants. The cell populations analyzed included mDCs, pDCs, monocyte subsets (classical, intermediate, and nonclassical monocytes), and Tfh cells. Circulating Tfh cells expressing activation markers (CXCR3^+^ICOS^+^PD-1^+^) were quantified at baseline to assess their role in supporting antibody responses (28).

#### Transcriptomic profiles.

RNA-Seq was conducted on nasal swabs from 121 participants and blood samples from 93 participants collected before LAIV to generate transcriptomic profiles following the protocol detailed in our previous work (8). Briefly, Gene Set Enrichment Analysis (GSEA) was performed using the fgsea Bioconductor package, ranking genes by their Spearman’s correlation coefficients between rlog-normalized expression and LAIV viral loads. Enrichment was assessed separately for Reactome pathways and a cell-subset marker set (50 defining genes per subset), and single-sample GSEA was also conducted using prevaccination (baseline) gene expression values for each participant. Normalized enrichment scores, adjusted *P* values, and leading-edge genes were extracted for each pathway. Pathways with an adjusted *P* value less than 0.1 were considered significant, representing a more stringent threshold than the commonly used *P* value less than 0.25.

#### Demographic and clinical data.

Detailed demographic data, including age, sex, nutritional status (weight-for-height *z* score), and health history, were collected for all 244 study participants to assess potential correlations with immune responses. Participants were monitored for adverse events, and any respiratory illnesses occurring during the study period were documented to evaluate safety and potential confounding factors.

### Data integration and preprocessing

The integrated dataset was generated using the standard extract-transform-load procedure, as described previously (17). Briefly, data from 6 primary datasets, each provided in CSV format and encompassing various immunological assays and demographic information, were integrated using the unique identifier Subject ID. This integration was facilitated by a custom *combine_data* function, which merged the datasets into a single comprehensive dataset. Data were obtained before vaccination (day 0) and on day 21 after vaccination for all measured parameters, including cellular, humoral, and mucosal values. Fold-changes were then calculated to obtain the LAIV-responsiveness measures, capturing both the preexisting immune state and the vaccine-induced responses. Before analyzing the integrated dataset, we performed several preprocessing steps. The proportion of missing values varied from 1% to 56% across features. We addressed these missing values using a median-based imputation (*medianImpute*), in which the median value of the corresponding feature replaced each missing entry. The data were then normalized by centering (subtracting the mean) and scaling (dividing by the standard deviation) of each feature. Features exhibiting zero variance (*zv*) and near-zero variance (*nzv*) were identified and removed to reduce noise and improve computational efficiency. Additionally, features with pairwise Pearson correlation coefficients greater than 0.85 were considered highly correlated and were filtered by retaining only 1 representative feature from each correlated group. The final dataset included a comprehensive set of immunological and demographic features representing various aspects of the immune response to LAIV.

### Data-driven immunogenicity responders subtyping

In this section, we describe the methodology used for clustering a dataset based on *t*-SNE dimensionality reduction (46), KNN graph construction, and Louvain community detection (47, 48). We also outline the optimization steps for selecting the best clustering result based on multiple clustering evaluation metrics.

#### t-SNE dimensionality reduction.

**X** ε *Rn × d* represents the dataset with *n* samples and *d* features. We first applied *t*-SNE to project the dataset into a lower-dimensional space, **Y** ε *Rn ×* 2. The *t*-SNE method aims to minimize the Kullback-Leibler (KL) divergence between probability distributions of points in high-dimensional and low-dimensional spaces. The objective function minimized by *t*-SNE is:

(Equation 1)
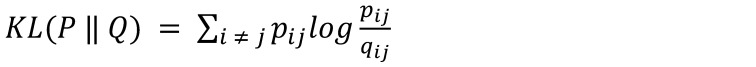


where *p_ij_* is the similarity between points *i* and *j* in the high-dimensional space, and *q_ij_* is the similarity in the low-dimensional space.

#### KNN graph construction.

Given the *t*-SNE projection Y, we constructed a KNN graph to capture the local structure of the data. For each point *i*, the *k* nearest neighbors are determined based on the Euclidean distance in the 2D space:

(Equation 2)
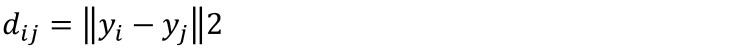


where y*i* and y*j* are the *t*-SNE coordinates of points *i* and *j*, respectively. The graph *G* = (*V, E*) is constructed with *V* being the set of nodes (samples) and *E* the set of edges connecting each point to its *k* nearest neighbors. The weight of each edge is defined as:

(Equation 3)
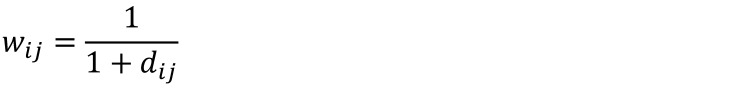


where smaller distances lead to higher edge weights, emphasizing closer neighbors.

#### Louvain clustering for community detection.

The Louvain method is applied to the KNN graph for community detection. The Louvain algorithm optimizes *modularity Q*, which measures the density of edges within communities compared with what would be expected in a random graph. The modularity is defined as:

(Equation 4)
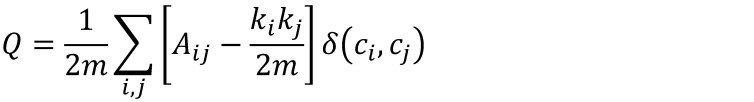


where: *Aij* is the adjacency matrix of the graph, *ki* is the degree of node *i*, *m* is the total number of edges, *ci* is the community assignment of node *i*, and δ(*ci, cj*) is the Kronecker delta function that equals 1 if *ci* = *cj* and 0 otherwise.

The Louvain method iteratively maximizes *Q* by merging nodes and communities to achieve an optimal partitioning.

#### Iterative optimization of clustering resolution.

To explore different clustering resolutions, we applied the Louvain algorithm over a range of resolutions *r*. The resolution *r* controls the granularity of the clustering, with lower resolutions favoring fewer, larger clusters, and higher resolutions producing more, smaller clusters. We define a sequence of resolutions {*r*_1_, *r*_2_..., *r_k_*} such that *r*_i+1_ = *r*_i_ + Δ*r*, Δ*r* = 0.1 for each iteration *i*. For each resolution *r_i_*, we computed the modularity *Q*(*r_i_*) and the number of clusters *C*(*r_i_*). We kept the clustering results that fell within the desired range of cluster counts: *C*_min_ ≤ *C*(*r_i_*) ≤ *C*_max_.

#### Evaluation metrics for best clustering selection.

After obtaining multiple clustering results across different resolutions, we selected the best result based on a combination of metrics. Modularity Q — we aim to maximize the modularity score, which indicates better separation of communities. Silhouette score S — the silhouette score measures the cohesion and separation of clusters. For each point *i*, the silhouette score is defined as:

(Equation 5)
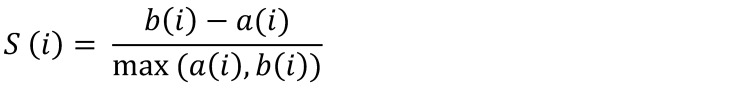


where *a*(*i*) is the average distance between *i* and all other points in the same cluster, and *b*(*i*) is the average distance between *i* and all points in the nearest cluster. We maximized the average silhouette score across all points.

The Davies-Bouldin index (DBI) is computed as:

(Equation 6)
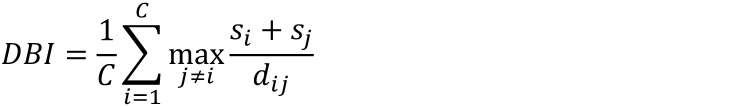


where *s_i_* is the average distance within cluster *i*, and *d_ij_* is the distance between cluster centroids *i* and *j*. A lower DBI indicates better clustering. A lower DBI indicates better clustering.

The Calinski-Harabasz index (CH) is given by:

(Equation 7)
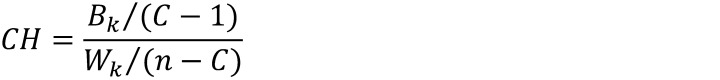


where *B_k_* is the between-cluster dispersion and *W_k_* is the within-cluster dispersion. Higher CH values indicate better clustering.

#### Combined score for clustering selection.

For each clustering result, we normalized the metrics and computed a combined score *M* to select the best clustering: *M* = α1 × normalize(*Q*) + α2 × normalize(*S*) + α3 × (1 *–* normalize[*DBI*]) + α4 × normalize(*CH*), where α1*,* *α*2*,* *α*3*,* and *α*4 are weights assigned to each metric, and the normalization function scales each metric to the range [0, 1]. The clustering result with the highest score, *M*, was selected as the final optimal clustering.

#### Predictive modeling of immunophenotypic clusters.

After clustering, the immunophenotypic groups identified in Immunaut’s first step were treated as categorical outcomes in a predictive modeling framework. In this second step, the SIMON platform (17, 31) was employed to systematically evaluate 141 machine learning algorithms, aiming to discover a minimal set of baseline features capable of accurately predicting immunophenotypic group membership. Predictors were baseline measurements of immune and molecular features, with immunophenotypic groups from clustering serving as the outcome variable. Data preprocessing procedures included centering and scaling, median imputation for missing values, removal of highly correlated features, and zero- and near-zero-variance filtering to ensure data quality. The dataset was divided into 80% training and 20% testing sets for model development, allowing for independent model validation. Parallel computation was implemented to expedite the training and selection process, with the number of cores for parallel processing set to the number of available CPU cores minus 1. Model evaluation during training utilized a 10-fold cross-validation approach, repeated 3 times to enhance robustness and mitigate overfitting. The performance of each model was assessed on the independent test set, using a confusion matrix and AUC metrics to provide unbiased evaluations of predictive accuracy across the 3 response classes. One-versus-all receiver operating characteristic curves were generated for each class using the *pROC* package in R, allowing for a detailed assessment of model sensitivity and specificity. To gain insights into feature significance, variable importance scores were calculated for each model within each response class. These scores were aggregated across classes to highlight baseline features with the highest predictive power, providing a comprehensive view of the immune and molecular markers most strongly associated with specific immunophenotypic group memberships.

### Model interpretability

SHAP analysis was conducted using the DALEX (moDel Agnostic Language for Exploration and eXplanation) package in R (https://github.com/ModelOriented/DALEX/) to interpret the contribution of individual features to the gradient boosting machine model’s predictions for each LAIV responder group (49, 50). SHAP values were computed to quantify the local, observation-specific impact of each feature on the model’s output, providing an additive decomposition of predictions into contributions from individual features and an intercept term. For each observation, SHAP values reflect how much each feature increases or decreases the predicted probability of belonging to a specific cluster (group 1: CD8^+^ T cell responders, group 2: mucosal responders, group 3: systemic, broad influenza A responders) relative to the baseline prediction (intercept). The analysis was implemented by linking the trained gradient boosting machine model with the DALEX explainer function, generating SHAP values for features prioritized by global variable importance scores. Feature contributions were visualized for each cluster using horizontal bar plots, where the magnitude and direction of SHAP values indicate the relative importance and influence (positive or negative) of each feature on the prediction. This approach provided granular insights into how baseline immune features drive LAIV immunogenicity across different responder groups.

#### Tree-based analysis.

All analyses were conducted in R using the rpart and rpart.plot packages. Data were loaded from a CSV file and merged with feature mapping information to restore original feature names. Missing values were replaced by column medians to ensure complete datasets for model fitting. Categorical variables were converted to factors, and continuous variables were discretized into meaningful bins based on predefined cutoffs. After discarding redundant variables, a decision-tree model was fitted using *rpart* with parameters set to ensure appropriate pruning (cp = 0.01) and sufficient sample sizes for splits (minsplit = 70, minbucket = 10). The tree was visualized with *rpart.plot*, and its full rule set was extracted using rpart.*rules* and saved for downstream interpretation.

### Data analysis

Statistical analysis was performed using R (https://www.r-project.org/) package ggpubr version 0.4.0. Integrative and machine learning analysis, including hierarchical clustering, *t*-SNE, KNN, and Louvain clustering, and supervised machine learning approach SIMON, were performed using PANDORA software version 0.2.1. All data visualizations were conducted in R version 4.3.1 with the tidyverse package (version 2.0.0) for data wrangling. Heatmaps were created using the pheatmap package (version 1.0.12), polar plots were produced with ggplot2 (version 3.5.1) and the Wes Anderson color palette (version 0.3.7), and radar plots were generated with fmsb (version 0.7.6). Scaled median pathway expression values were calculated by grouping genes by pathway, omitting any missing values, and computing the median for each pathway-group pair. These scaled median values were used in all visualization techniques for consistent metric comparison across clusters in each plot type. Feature-specific polar plot values were further transformed using log_10_ to control significant variances, ensuring a more balanced visualization of expression levels across features.

### Data availability

Data values reported in this manuscript are provided in the Supporting Data Values file. The complete, integrated, and deidentified dataset supporting the findings in this study is available on Zenodo (51): Comprehensive Multimodal Immune Response Dataset for LAIV Vaccination in Pediatric Cohorts. This dataset includes all baseline and postvaccination measurements required to reproduce the analyses presented in this study. In addition, deidentified, processed/normalized gene expression data for baseline nasal and blood RNA-Seq for all participants are available on Zenodo (52). Researchers requiring access specifically to raw data should contact the corresponding author to initiate a request. Access will be facilitated through a formal data transfer agreement managed by London School of Hygiene and Tropical Medicine to ensure compliance with ethical approvals. The Immunaut platform, used for mapping immune profiles and predicting vaccine responses, is accessible via the PANDORA AI platform (https://pandora.atomic-lab.org/) and as an R package on CRAN (https://cran.r-project.org/web/packages/Immunaut/index.html). General documentation for the Immunaut package is hosted on GitHub (https://github.com/atomiclaboratory/Immunaut). Furthermore, to ensure reproducibility of our specific findings, the exact code used for figure generation and modeling presented in this work has been deposited on GitHub (https://github.com/atomiclaboratory/Immunaut/tree/master/R-package#example-5-using-immune-response-dataset-for-laiv-vaccination-in-pediatric-cohorts-dataset).

### Study approval

Written informed consent was obtained from parents or guardians, and the study was approved by The Gambia Government, the UK Medical Research Council Joint Ethics Committee, and the Medicines Control Agency of The Gambia, adhering to the International Conference on Harmonisation Good Clinical Practice standards.

## Author contributions

IT and SH are co–first authors.The order was done alphabetically by last name. IT and AT conceptualized the study. IT, SH, YJJ, BBL, KH, CP, AM, JMCQ, KS, and AT devised the methodology. IT, SH, and AT conducted the investigation. IT, AT, YJJ, BBL, and TIDS curated the data. IT was responsible for software. IT, AT, and SH were responsible for visualization. AT acquired funding, provided project administration, and supervised the study. IT, SH, and AT wrote the original draft. IT, SH, BBL, YJJ, KH, AM, JMCQ, PM, KS, FK, BK, DB, CP, AGCM, HN, TIDS, and AT reviewed and edited the manuscript.

## Supplementary Material

Supplemental data

ICMJE disclosure forms

Supplemental table 1

Supplemental table 3

Supporting data values

## Version 1 07/15/2025

In-Press Preview

## Version 2 09/16/2025

Electronic publication
