# Supplementary material for: Integrative mapping of preexisting influenza immune landscapes predicts vaccine response
Source: J Clin Invest. 2025 Jul 15;135(18):e189300. doi: 10.1172/JCI189300 (PMC12490229; doi:10.1172/JCI189300)
Supplement: Supplemental data [file jci-135-189300-s323.pdf]

Supplemental materials for *"Integrative mapping of pre-existing influenza immune landscapes predicts vaccine response"*

Authors: Stephanie Hao[1], Ivan Tomic[1], Benjamin B. Lindsey[2, 3], Ya Jankey Jagne[4], Katja Hoschler[5], Adam Meijer[6], Juan Manuel Carreño Quiroz[7,8], Philip Meade[7,8], Kaori Sano[7,8], Chikondi Peno[9], André G. Costa-Martins[10,11], Debby Bogaert[9, 12], Beate Kampmann[13,14], Helder Nakaya[10,15], Florian Krammer[7,8,16, 17], Thushan I. de Silva[2, 3, 12] and Adriana Tomic[1, 18, 19]

Supplemental figures

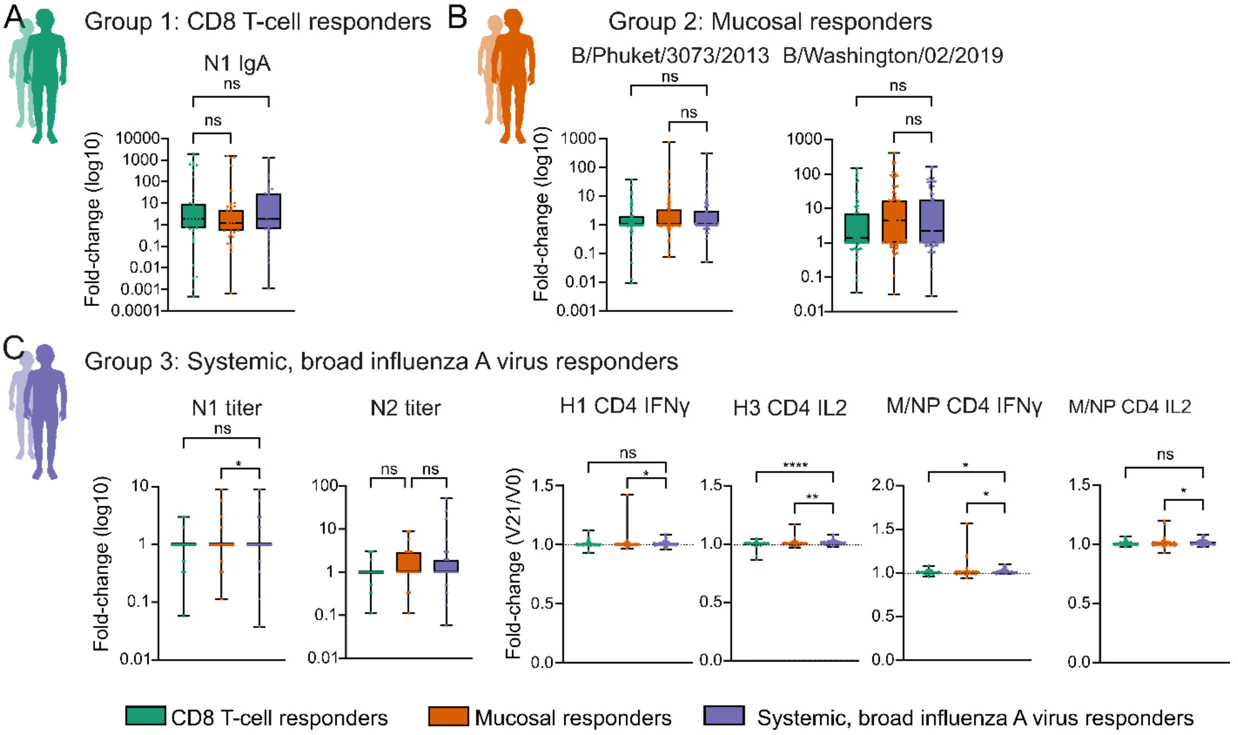

Supplemental Figure 1. Differential immune responses across LAIV-induced responder groups. (A) Group 1: CD8 T-cell responders (green) distinctive feature shown as box plot fold-change in N1-specific IgA levels (log10). **(B)** Group 2: Mucosal responders (orange) show fold-changes in influenza B virus-specific IgG levels (log10) (B/Phuket/3073/2013, B/Washington/02/2019) across groups. **(C)** Group 3: Systemic, broad influenza A virus responders demonstrate fold-changes (log10) in titers of antibodies binding to N1 and N2, and fold-change CD4 T-cell cytokine responses (IFNγ and IL2) to influenza A virus hemagglutinin (H1 and H3) and matrix/nucleoprotein antigens (M/NP). Box plots denote min to max values, and points are all individuals within the group, with significance levels calculated using one-way ANOVA Kruskal-Wallis test with Dunn's multiple comparison test to adjust for multiple testing. Significance is indicated as follows: ns = not significant, *p < 0.05, **p < 0.01, ***p < 0.001, ****p < 0.0001.

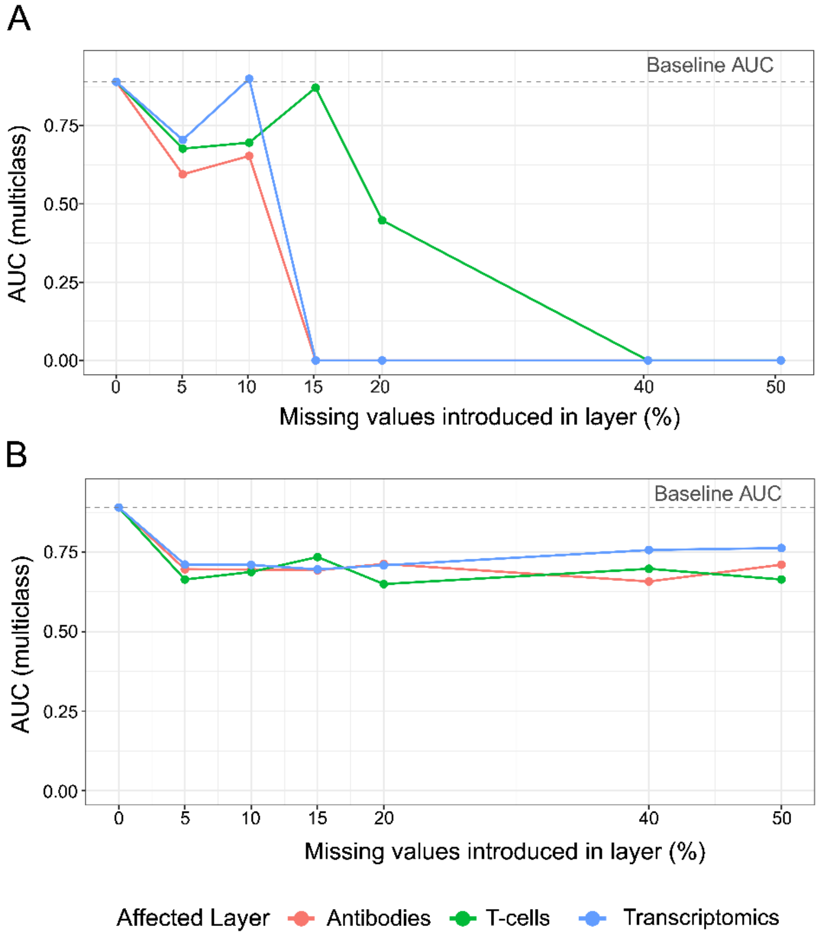

Supplemental Figure 2. Robustness of the multi-omics integration model to missing values in individual data layers. Assessment of Gradient Boosting Machine (gbm) model performance sensitivity to missing data introduced systematically into individual input layers. Multiclass Area Under the Curve (AUC) is plotted against the percentage of features randomly set to missing within a specific data layer (Antibodies - red, T-cells - green, Transcriptomics - blue), ranging from 0% to 50%. The horizontal dashed line indicates the baseline multiclass AUC achieved by the gbm model trained on the original data (0% additional missing values). The analysis compares two preprocessing strategies: **(A)** gbm model performance when no imputation step is applied to handle the introduced missing values prior to model training. **(B)** gbm model performance when missing values introduced within the affected layer are imputed using the median value of the respective feature (across samples) prior to model training.

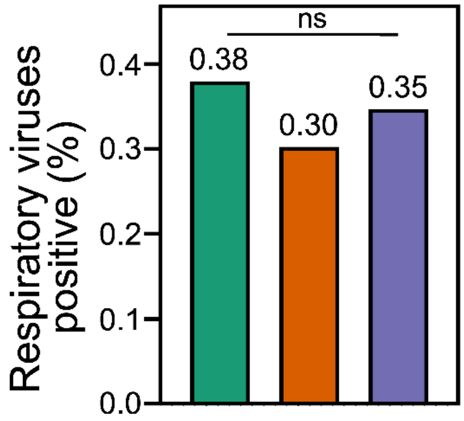

Supplemental Figure 3. Proportion of children positive for asymptomatic respiratory viruses before vaccination, stratified by post-vaccination response phenotype. Bars represent the percentage of children testing positive within the CD8 T-cell responders group (green bar, 38%), Mucosal responders group (orange bar, 30%), and Systemic, broad influenza A virus responders group (purple bar, 35%). Differences in these pre-vaccination positivity rates among the defined response groups were not statistically significant (ns; $p = 0.57$, Chi-squared test).

Tables

Supplemental Table 1. *Available online.*

Supplemental Table 2. Performance comparison of the gbm model using different input data modalities. This table presents the performance metrics of the Gradient Boosting Machine (gbm) model when trained and evaluated using different subsets of the data. Performance is shown for models trained on individual data modalities (Antibodies only, T-cells only, Transcriptome only), combinations of modalities, and the fully integrated dataset. Metrics reported include Accuracy, AUC, Kappa, F1 Score, Negative Predictive Value, Positive Predictive Value, Specificity, and Sensitivity, allowing for comparison of the predictive power contributed by each data type.

Model input data	Accuracy	AUC	Kappa	F1 Score	Neg Pred Value	Pos Pred Value	Specificity	Sensitivity
Antibodies only (HAI + IVPM)	0.5319	0.6662	0.298	0.442	0.7211	0.4466	0.7186	0.438
T-cells only	0.4468	0.6467	0.1596	0.3539	0.6738	0.34	0.6723	0.3403
Transcriptome only	0.4255	0.5889	0.112	0.3958	0.6904	0.4063	0.6892	0.3772
Transcriptome + Antibodies	0.5319	0.6229	0.2985	0.4197	0.7084	0.4145	0.7047	0.4066
Transcriptome + T-cells	0.5106	0.6706	0.2529	0.3612	0.6678	0.3077	0.6664	0.3278
Integrated data	0.6383	0.8182	0.4594	0.7097	0.9	0.6471	0.8333	0.7857

Supplemental Table 3. *Available online.*
